# Investigating Functional and Folding Stability of an Engineered *E. coli* L-asparaginase Harboring Y176F/S241C Mutations

**DOI:** 10.34172/apb.2024.048

**Published:** 2024-06-22

**Authors:** Mahrokh Dastmalchi, Maryam Hamzeh-Mivehroud, Hassan Rezazadeh, Mohammad M Farajollahi, Siavoush Dastmalchi

**Affiliations:** ^1^Department of Medical Biotechnology, Faculty of Allied Medical Sciences, Iran University of Medical Sciences, Tehran, Iran.; ^2^Biotechnology Research Center, Tabriz University of Medical Sciences, Tabriz, Iran.; ^3^School of Pharmacy, Tabriz University of Medical Sciences, Tabriz, Iran.; ^4^Pharmaceutical Analysis Research Center, Tabriz University of Medical Sciences, Tabriz, Iran.; ^5^Faculty of Pharmacy, Near East University, POBOX:99138, Nicosia, North Cyprus, Mersin 10, Turkey.

**Keywords:** L-asparaginase, Enzyme parameters, Thermodynamic stability

## Abstract

**Purpose:**

L-asparaginase has been widely recognized as a critical component in the treatment of various types of lymphoproliferative disorders, since its introduction in 1960s. However, its use in some cases leads to allergic reactions rendering the continuation of treatment unfeasible. Thus, the development of L-asparaginase from alternative sources or the production of engineered enzymes have always been considered. This study aimed to produce and evaluate a novel enzyme designed based on the sequence of L-asparaginase from *Escherichia coli* bacteria with Y176F/S241C mutations.

**Methods:**

The Y176F/S241C mutant L-asparaginase was successfully expressed as the GST-fusion protein in *E. coli*, and then was subjected to affinity and size exclusion chromatography. The activity of the purified enzyme was determined based on the released ammonia as the result of substrate hydrolysis using Nessler’s reagent. Chemical denaturation experiment in the presence of increasing concentration of guanidinium chloride was applied to determine the folding stability of the purified enzyme.

**Results:**

The mutant enzyme was purified with an efficiency of 77-fold but at a low recovery of 0.7%. The determined kinetic parameters Km, Vmax, kcat, specific activity and catalytic efficiency were 13.96 (mM), 2.218 (mM/min), 273.9 (min^-1^), 237.8 (IU/mg) and 19.62 (mM^-1^ min^-1^), respectively. Moreover, unfolding free energy determined by guanidinium chloride induced denaturation for mutated and commercial L-asparaginase enzymes were 8421 J/mol and 5274 J/mol, respectively.

**Conclusion:**

The mutant enzyme showed improved stability over the wild-type. Although the expression level and recovery were low, the mutant L-asparaginase demonstrated promising activity and stability, with potential clinical and industrial applications.

## Introduction

 L-asparaginase, a type of aminohydrolase enzyme (EC 3.5.1.1), is responsible for hydrolyzing the amino acid L-asparagine into L-aspartic acid and ammonia. This enzyme has been recognized as a key therapeutic agent for the treatment of acute lymphoblastic leukemia (ALL) and Hodgkin’s lymphoma over the past few decades.^1,2^ L-asparaginase also exhibits therapeutic potential in treating other blood and non-blood diseases, including pancreatic cancer, acute myeloid leukemia, and lymphosarcoma.^3^ The food industry also employs L-asparaginase to prevent the formation of acrylamide during high-temperature food processing. The presence of acrylamide in carbohydrate-rich foods, such as fried potatoes, chips, and roasted coffee beans, poses a significant health risk as it enters the food chain as a byproduct.^4^

 Since its introduction to the clinic, L-asparaginase proved remarkably effective with 93% complete remission in pediatric patients diagnosed with ALL.^5^ Part of this improved treatment was brought about by the use of chemically modified form of L-asparaginase covalently conjugated to polyethylene glycol to decrease immunogenicity of the enzyme and prolong its half-life.^6,7^

 Like all drugs, L-asparaginase can cause adverse reactions and side effects.^8,9^ Since the L-asparaginase available in the market originates from bacterial sources, it can trigger the production of anti-asparaginase antibodies, causing allergic reactions leading to a variety of side effects, ranging from simple allergic reactions to more severe life-threatening consequences.^10^

 Another problem displayed by commercial L-asparaginase is a secondary L-glutaminase activity, but the role of this activity is not fully understood and the literature contains conflicting evidence regarding its effects.^11^ There is evidence that L-glutaminase activity is not required for *in vivo* tumor cytotoxicity and that reducing L-glutaminase activity leads to fewer side effects,^12^ while some reports suggest that L-glutaminase co-activity is necessary to potentiate the anti-tumor effect in cancer cells and is even indispensable for the enzyme’s antitumoral properties.^13^ However, since L-glutamine is the most abundant amino acid in the blood and plays a crucial role in many biosynthetic reactions, its deprivation has been linked to several deleterious effects, including hepatotoxicity, pancreatitis, hyperammonemia, neurotoxicity, hyperglycemia, leukopenia, and coagulation abnormalities such as thrombosis and hemorrhage in patients receiving L-asparaginase treatment.^14^

 Resistance to L-asparaginase treatment involves various mechanisms. For example, asparagine and glutamine starvation can lead to the induction of asparagine synthetase and glutamine synthetase enzymes, which subsequently can rescue cancer cells from apoptosis caused by L-asparaginase. Therefore, reducing serum asparagine, which prevents the proliferation of leukemic cells, is a critical element in preventing relapse.^15^

 Currently, L-asparaginase from bacteria such as *Escherichia coli* and *Erwinia chrysanthemi* are employed, but as stated before, the enzyme derived from these microorganisms often presents issues like hypersensitivity and immunosuppression.^16^ Eukaryotic microorganisms like filamentous fungi and yeasts have been also investigated for enzyme production in hope of alleviate some of the issues associated with bacterial L-asparaginase.^17^

 L-asparaginase is regarded as the gold-standard therapy of childhood ALL and also proved effective in adult patients. However, its effectiveness has been scrutinized due to the range of side effects experienced by the patients treated with it.^18,19^ The rapid plasma elimination, the need for multiple administrations, frequent allergic reactions, and thrombotic complications are among major disadvantages of L-asparaginase.^20,21^ Hence, the search for new L-asparaginase with possibly improved enzymatic properties has been the focus of numerus studies for a long time. A new biotechnology-based pharmaceutical product with improved properties is introduced as a bio-superior of an existing biopharmaceutical.^22-24^ These improved properties include affinity, selectivity, catalytic activity, and stability against degradation. For example, L-asparaginase derived from *E. chrysanthemi* was approved by FDA in 2011 to be used by those patients showing hypersensitivity to *E. coli*-derived L-asparaginase.^25^

 Improving the therapeutic properties of L-asparaginase is critical for enhancing the treatment success of ALL. Studies by Mehta et al showed that the Y176F mutation of *E. coli* L-asparaginase results in significantly increased apoptosis in lymphocytes of ALL patients. Furthermore, the glutaminase activity in Y176F mutant enzyme is significantly reduced.^15^ The structural similarity between L-asparagine and L-glutamine likely explains the enzyme’s glutaminase activity.^26^ Studies have indicated that the Y176F mutation increases enzyme efficiency, as demonstrated by the increased V_max_/K_M_ for L-aspartic acid beta hydroxamate.^27^ Another mutation that has beneficial effects on L-asparaginase performance is S241C.^28^ Mutations K107L, S241C, and R269F not only enhance stability but also reduce the toxicity of L-asparaginase. Additional studies showed that mutations such as L1G, K107L, S241C, and R269F result in non-toxic enzymes with greater stability and a longer half-life in the body. The glutaminase activity of various mutants of *E. coli* L-asparaginase at position 176 (Y176F, Y176S, and K288S/Y176F) is significantly lower than the wild type. Variants Y176F and W66Y demonstrate high cytotoxicity in leukemic cell lines.^15^

 In our previous investigation, we analyzed numerous reports on *E. coli* L-asparaginase mutations and their consequences on stability, function, toxicity, and other characteristics of the enzyme and proposed, using in silico simulation, that a dual Y176F/S241C mutant of *E. coli* L-asparaginase may possess significantly improve stability over wild type enzyme. Furthermore, it was shown experimentally that the mutant enzyme retains enzymatic activity.^5^ Except for our prior preliminary study, there is no empirical data on simultaneous effect of these two mutations on *E. coli* L-asparaginase. The aim of the current work is the production of Y176F/S241C double mutant recombinant *E. coli* L-asparaginase enzyme and investigate its enzymatic and stability properties. The results could be used for the production of L-asparaginase with high efficacy, low L-glutaminase activity, improved stability, and perhaps with better therapeutic properties.

## Materials and Methods

###  Chemicals

 All reagents were of analytical grade. Commercial *E. coli* L-asparaginase (Bionase®) was from ZYDUS Pharma Ltd (India). Tryptone, yeast extract, isopropyl-β-D-thio galactopyranoside (IPTG), Triton X-100, trypsin, agar, glycerol, phenylmethylsulfonyl fluoride (PMSF), N,N,N’,N’-tetramethyl ethylene diamine (TEMED), and anhydrous D-glucose, were acquired from AppliChem (Darmstadt, Germany). Mini-prep plasmid extraction kit was purchased from SinaClon (Iran). Maxi and Mini prep plasmid extraction kit were purchased from QIAGEN (Hilden, Germany). Taq 2X Master Mix Red was from Ampliqon (Denmark). BamH1, EcoR1, and T4 DNA ligase was from Thermo Fisher Scientific (USA). Ni Sepharose^TM^ 6 Fast flow medium and Glutathione Sepharose^TM^ 4B medium were from GE Healthcare Life Sciences (Sweden). All solutions were prepared using ultrapure water obtained from Milli-Q® Gradient water purification system (Millipore Corporation, Bradford, MA, USA). Primers were supplied by Bioron (Ludwigshafen, Germany).

###  Subcloning, expression and purification of mutant L-asparaginase

 To prepare the plasmids B and C, the original plasmid (plasmid A) harboring mutant L-asparaginase (L-ASPmut-pET-22b( + )) was utilized.^5^ PCR amplification was performed using 2 sets of primers (Table 1) designed based on plasmid A sequence. PCR reaction in the presence of primers F1 and R and the DNA template (plasmid A) were used to amplify the coding gene for the mutant L-asparaginase as well as the upstream 6 × HIS tag, while using F2 and R primers, the PCR product has only coding gene for the mutant L-asparaginase. The plasmids expressing the mutant L-asparaginase N-terminally fused to glutathione S-transferase (GST) were constructed by inserting BamHI and EcoRI double-digested PCR products between the same restriction sites in pGEX-6p-1 vector. The accuracy of the constructs was verified by sequencing. All PCR reactions were performed using Pfu DNA polymerase (Bioron, Germany) under temperature program consist of a 3 minutes initial denaturation step at 95 °C, followed by 35 cycles of denaturation (95 °C for 30 seconds), annealing (60 °C for 60 seconds) and extension (72 °C for 90 seconds) steps, and a final extension step at 72 °C for 6 minutes.

**Table 1:** Oligonucleotide primers used for amplification of mutant L-asparaginase

**Plasmid **		**Sequence of primer **
Plasmid B	Forward (F1)	5’- CCA ***GGATCC*** ATGGCCATGGGGCAT-3’
Plasmid C	Forward (F2)	5’- GAA ***GGATCC*** ATGGAGTTTTTCAAAAAGACGGC-3’
Both plasmides	Reverse (R)	5’- GCTC ***GAATTC*** GGTACCCTAGTACTGATTGAAG-3’

The bold and italic regions in the sequence of primers are restriction sites. ***GGATCC*** in F1 and F2 forward primers is *Bam*H1 restriction site, and ***GAATTC*** in R reverse primer is *Eco*R1 restriction site.

 Plasmids containing GST-L-ASPmut coding sequence were transformed and expressed in different *E. coli* strains (BL21(DE3), pLysS and Origami cells). Bacteria were grown at 37 °C in one liter of LB broth containing 100 µg/mL ampicillin to an optical density of 0.7-0.9 and expression of the GST-L-ASPmut protein induced by addition of 1 mM of isopropyl-1-thio-ß-D-galactopyranoside (IPTG) followed by overnight incubation at 20 °C. Cells were harvested and resuspended in lysis buffer (50 mM Tris-HCl pH 7.2, 500 mM NaCl, 10% Triton-X100, 1.4 mM phenyl methyl sulfonyl fluoride (PMSF), 0.1% beta-mercaptoethanol (2-ME), 0.1 mg/mL lysosome, 10 μg/mL DNase I). Cell disruption was induced by 3 rounds of freeze-thaw cycles coupled with 5 rounds of sonication pulses (60% amplitude) for 30 seconds with a pause interval of 30 seconds. The samples were cooled to 4 °C prior to sonication and kept on ice during sonication. The bacterial lysate was centrifuged at 13 000 rpm for 20 minutes at 4 °C. The supernatant was subjected to affinity chromatography by incubation with glutathione-Sepharose^TM^ 4B beads for 2 hours at 10 °C. Subsequently, the beads were washed with 5 column volumes of cleavage buffer (150 mM NaCl, 50 mM Tris pH 7, 1 mM EDTA and 5 mM 2-ME). To prepare recombinant protein free from GST, the beads with the bound GST-L-ASPmut protein was incubated with PreScission protease in cleavage buffer at 10 °C for 4 hours. Fractions containing L-ASPmut were collected and concentrated (Vivaspin® 500 Centrifugal Concentrator) and ultimately were subjected to size-exclusion chromatography using Sephacryl S-100 size-exclusion column (GE Healthcare). Proteins were eluted in 50 mM Tris pH 8.5, using a flow rate of 0.5 mL/min. Proteins at each step of the expression and purification were subjected to SDS-PAGE analysis. Protein concentration was measured by UV-Vis spectrophotometer at 280 nm, using extinction coefficients calculated from the amino acid sequence.

###  Enzyme activity, substrate specificity assay and Biochemical characterization of mutant L-asparaginase

 The enzyme activity was determined by measuring the released ammonia during L-asparagine hydrolysis using Nessler’s reagent.^29^ In order to reach temperature equilibration, the reaction mixture (2 mL) consisting of 50 mM Tris-HCl (pH 8.6) and 8.6 mM L-asparagine/L-glutamin was incubated at 37 °C for 5-6 minutes. After adding 100 µL from the sample containing purified L-ASPmut and/or commercial L-asparaginase at time zero, the reaction proceeded at 37°C for precisely 10 minutes and paused by adding 100 µL of 1.5 M trichloroacetic acid (TCA) only to “test” tubes. Prior to incubation, the blank tube was terminated by adding 100 µL of 1.5 M TCA. After centrifugation of the reaction mixture, 200 µL of clarified supernatant was mixed with 4.3 mL of ultrapure water and 0.5 mL of Nessler’s reagent and incubated at room temperature for 10 minutes. All the measurements were done by using UV-Vis spectrophotometer at 480 nm. Using the ammonium sulfate standard curve, the micromoles of the released ammonia were determined. The amount of enzyme that under the specified conditions such as the temperature of 37 °C and the pH of 8.6 is required to produce one micromole of the ammonia per minute is defined as one unit of the enzyme. Finally, the activity result for the purified L-ASPmut was compared to that of the commercial *E. coli* L-asparaginase (Bionaes®).

 In order to determine the kinetic parameters Vmax, Km and kcat of the purified mutant L-asparaginase, the enzymatic reaction was monitored at various concentrations of L-asparagine as a substrate (0, 4.3, 8.6, 17.2 mM) according to the above-mentioned spectroscopic method. The data were fitted to Michaelis–Menten equation by nonlinear regression using Prism software (version 8.4.3, GraphPad Software Inc.). The Michaelis-Menten constant K_M_ indicates the concentration of the substrate when the reaction rate is one-half of the maximal velocity (V_max_). The constant kcat (catalytic rate) shows the number of substrate molecules turned into the product by enzyme per second.^30^

###  Folding energy determination by chemical denaturation experiment 

 To study the folding energy of the produced and commercial enzymes, the changes in the intensity of the fluorescence emission of the enzymes were investigated in the presence of different concentrations of the denaturing agent guanidinium chloride (GdmCl). In these experiments, solutions with different concentrations of guanidinium chloride were prepared by serial dilution method. Then, by mixing certain volumes of enzyme and guanidinium chloride solutions, samples with constant enzyme concentration but variable concentrations of guanidinium chloride were prepared. The samples were excited at 280 nm and the emission spectrum was recorded in the range of 300 to 400 nm. Changes in emission intensity at 310 nm were used to determine the folding energy of enzyme.

## Results

###  Preparation of purified Y176F/S241C double mutant L-asparaginase (L- ASPmut )

 Two types of plasmids were designed and prepared to investigate the expression of the mutant L-asparaginase enzyme. The first plasmid, referred to as plasmid B, contains the coding sequence for the mutated L-asparaginase enzyme, 6 × HIS tag and cleavage sites for the PreScission and Factor Xa proteases in pGEX-6P-1 vector. The second plasmid, referred to as plasmid C, contains the coding sequence for the mutated L-asparaginase enzyme and the cleavage site for the PreScission enzyme again in pGEX-6P-1 vector.

**Figure 1:**
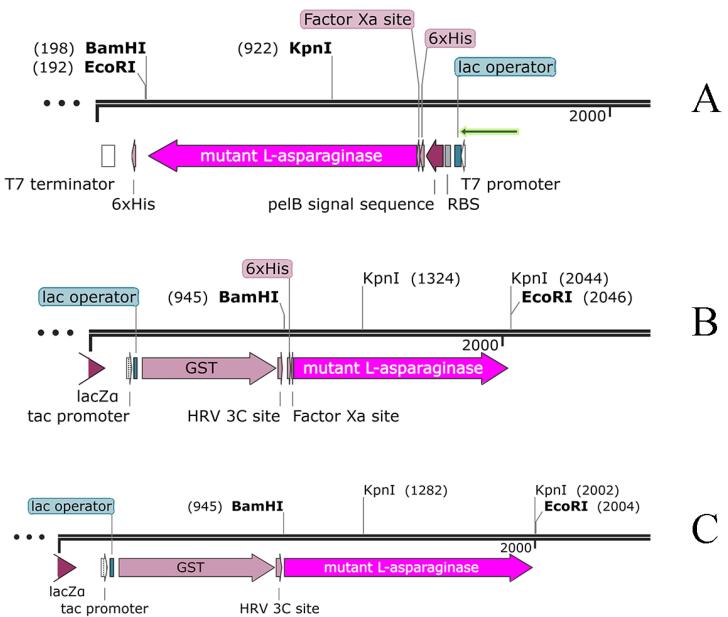
Genetic map of plasmids designed to express mutant L-asparaginase. Plasmid A is from previous study,^5^ Plasmids B and C were generated in this study. All plasmids are circular, which are displayed linearly for easy viewing. The sequence of the L-asparaginase gene is marked as a purple arrow with the name L-ASPmut

 The maps of the designed plasmids are illustrated in Figure 1. The prepared plasmids were transferred to the *E. coli* DH5α strain for amplification, and preliminary verifications using PCR, restriction enzyme digestion pattern and agarose gel electrophoresis (Figure S1 of Supplementary file 1). Finally, the correctness of the plasmids was confirmed by sequencing. The sequencing results for the plasmids B and C were translated into protein sequences, and compared to *E. coli* wild type L-asparaginase sequence (Accession Number: DB00023) using online pairwise global sequence alignment available at EMBL-EBI confirming the presence of Y176F/S241C mutations without any unwanted changes in the DNA sequence during the preparation of the genetic constructs plasmid B and C.

###  Expression and purification of mutant L-asparaginase

**Figure 2:**
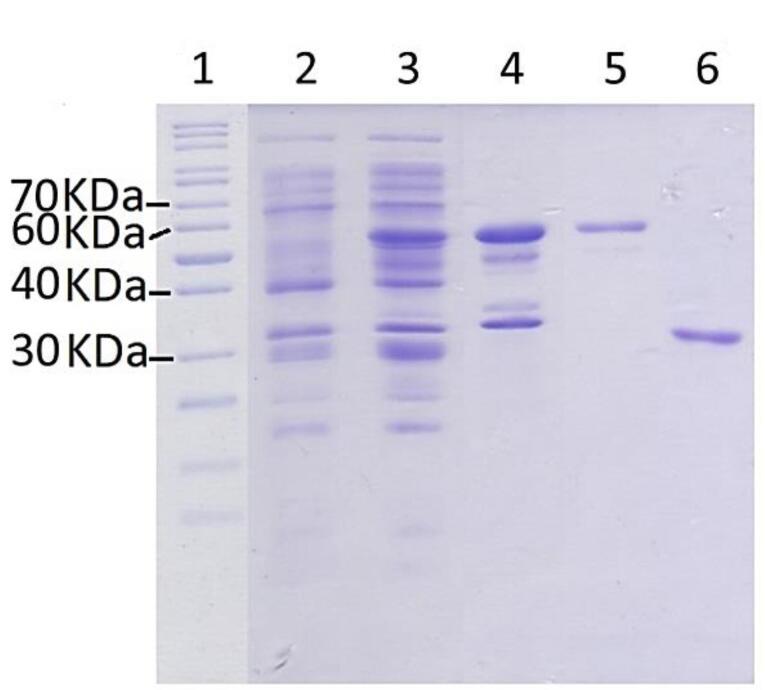
SDS-PAGE analysis of the produced Y176F/S241C mutant L-asparaginase protein from E. coli pLysS using plasmid C. The bands around ~70 kDa represents the L-asparaginase-GST fusion protein and bands between 40 and 30 kDa represents the L-asparaginase without the GST tag. Lanes 1 is protein weight marker. Lanes 2 and 3 are the total cell proteins before and after induction (final concentration of IPTG = 1mM), respectively. Lane 4 is the insoluble fraction. Lane 5 is glutathione-sepharose resin and the band at ~70 kDa represents the mutant L-asparaginase-GST fusion protein. Lane 6 shows the mutant L-asparaginase (at ~37 kDa), which is eluted from the fusion protein bound glutathione-sepharose resin by PreScission protease

 All three plasmids (plasmids A, B, and C) were used to transform different strains of *E. coli*, including BL21, Origami, and PlysS, and express the target protein. The bacterial cells were harvested from the culture and lysed, and then, the soluble fraction was separated and used for protein purification using affinity and size-exclusion chromatography methods. The soluble fraction from culture prepared using *E. coli *transformed by plasmid A, which contains a 6 × HIS sequence, was subjected to purification using immobilized metal ion affinity chromatography (IMAC) with nickel sepharose resin. However, the purification of the mutant recombinant L-asparaginase enzyme was not successful. The purification of the target protein using plasmid B, which contains both 6 × HIS and GST sequences, as well as cleavage sites for Factor Xa and PreScission proteases, by means of affinity chromatography with glutathione sepharose affinity column did not lead to successful result either. However, by transforming *E. coli* pLysS with plasmid C, and subsequent expression and purification, a required amount of mutant L-asparaginase was obtained and used in enzyme assay and protein folding experiments. Figure 2 displays the results of SDS-PAGE on the eluted sample from the glutathione-sepharose affinity resin under the influence of PreScission enzyme. The bands observed with weights of approximately 37 and 65.5 kilo Daltons correspond to the mutated L-asparaginase and GST-bound L-asparaginase, respectively.

**Figure 3:**
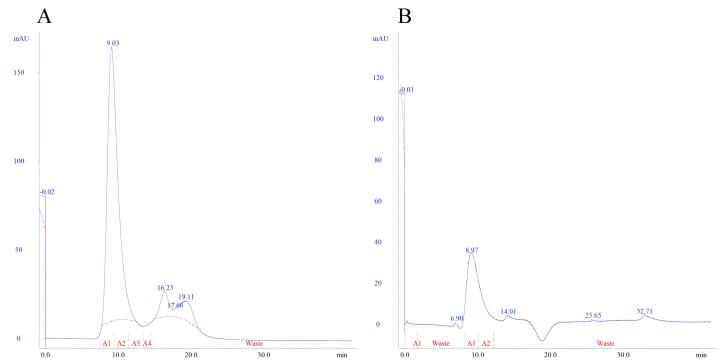
A representative size-exclusion chromatograms for the purification of (A) mutant L-asparaginase obtained from affinity purification and (B) commercial L-asparaginase

 After performing purification *via* affinity chromatography, the eluted protein sample was subjected to size-exclusion chromatography according to the condition described in the Materials and Methods section. The chromatograms in Figure 3 display the outcomes obtained for the mutant enzyme produced in this study and the commercial enzyme, respectively. The protein concentration for the samples during purification step was measured using UV spectrophotometry at 280 nm against a calibration curve (R^2^ = 0.997) prepared using BSA standard solutions.

###  Enzyme activity and kinetic parameters of the mutant L-asparaginase

 The enzyme activity was measured using Nessler’s assay according to the protocol outlined above. The obtained asparaginase and glutaminase activities for the purified mutant L-asparaginase were 237.8 ± 22.1 and 17.8 ± 2.5 (IU/mg ± SD), respectively, while the activities for the purified commercial enzyme were 167.8 ± 22.0 and 106.6 ± 3.8.

**Figure 4:**
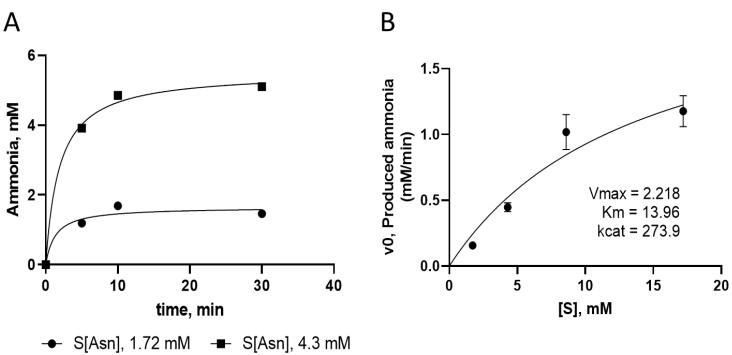
(A) Changes in the concentration of ammonia produced by the activity of the recombinant enzyme at different concentration of substrate (L-asparagine) over time. (B) Kinetic parameters analysis for recombinant enzyme and the corresponding Michaelis–Menten plot

 The assay was used to determine the enzyme kinetic parameters for the double mutated recombinant L-asparaginase. The changes in the concentration of ammonia produced by the activity of the recombinant enzyme over time at different concentrations of the substrate in Nessler’s reaction were used to determine initial velocity and kinetic parameters as shown in Figure 4.

###  Folding free energy of pure mutant and commercial L-asparaginase 

**Figure 5:**
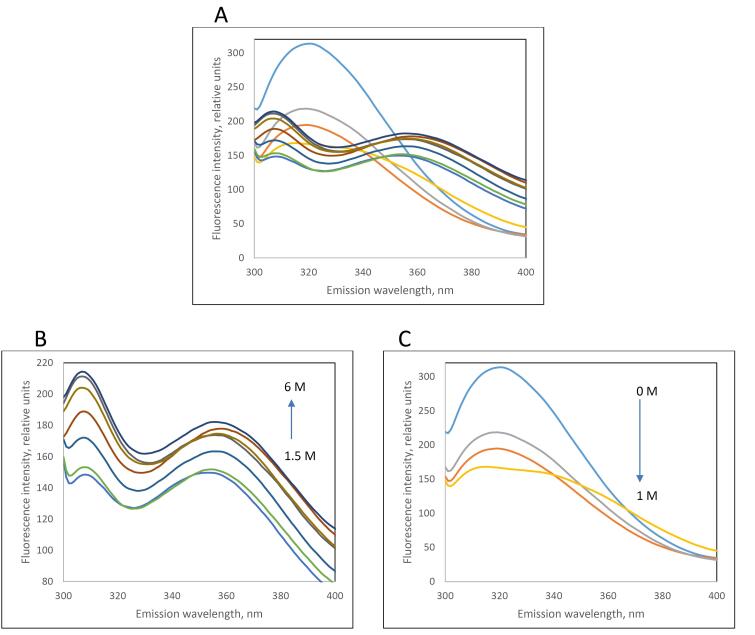
(A) Fluorescence emission spectra of purified recombinant L-asparaginase (with Y176F/S241C double mutations) with AC and SEC methods in the presence of different concentrations of guanidinium chloride (0 to 6 M). Due to changes in the pattern of fluorescence spectra at different concentration of GdmCl, they are separated in two graphs B and C

**Figure 6:**
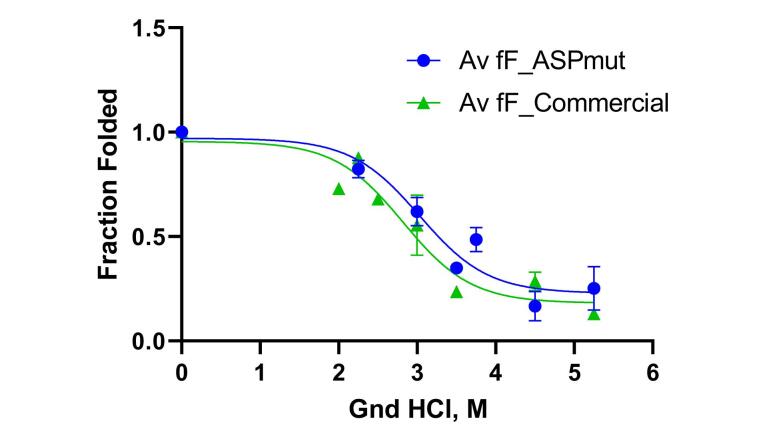
Folded fraction changes of designed L-asparaginase enzyme (with Y176F/S241C mutations) purified by AC and SEC methods (blue graph) and commercial enzyme in the presence of different concentrations of guanidinium chloride (green graph)

**Figure 7:**
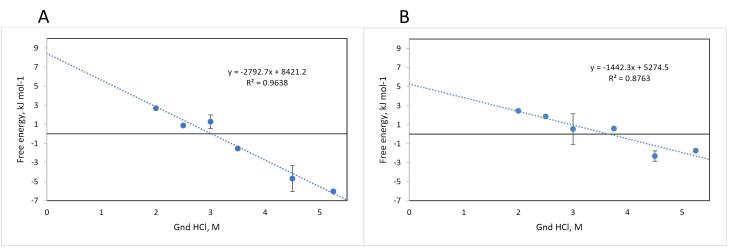
Folding free energy changes of designed L-asparaginase enzyme (with Y176F/S241C mutations) purified by AC and SEC methods (A) and commercial enzyme (B) in the presence of different concentrations of guanidinium chloride. The equations for the trend lines are in Jules

 The variation of fluorescence intensity of the designed recombinant L-asparaginase enzyme (with Y176F/S241C mutations) in the presence of different concentrations of guanidinium chloride (GdmCl) was examined, and related to the structural changes (unfolding) of the enzyme *** (***Figure 5 and Figure S2). The fractions folded/unfolded for the enzymes were determined based on the fluorescence intensities measured at different concentrations of GdmCl, as shown in Figure 6. The ratio of unfold and fold fractions of enzyme at each concentration of GdmCl was used to calculate equilibrium constant (*K*_eq_) and unfolding free energy change, or Gibbs free energy of unfolding at that concentration of GdmCl using the following equations: ∆G = RT ln*K*_eq_ where *K*_eq_ = [F]/[U]. By plotting the ∆G values against GdmCl concentration, the unfolding free energy of enzyme in the absence of denaturant (∆G_H2O_) can be determined using extrapolation method form the intercept of the line at zero concentration of GdmCl. The obtained Gibbs free energy of unfolding for mutated and commercial L-asparaginase enzymes were 8421 J/mol and 5274 J/mol, respectively (Figure 7).

## Discussion

 L-asparaginase (EC 3.5.1.1) is a crucial enzyme in the hydrolysis process that converts L-asparagine to L-aspartic acid, releasing a molecule of ammonia. This enzyme is a critical chemotherapy agent for various types of lymphomas, particularly ALL and Hodgkin’s lymphoma.^31,32^ L-asparaginase is produced using biotechnological methods, using sequences identified in *E. coli* and *E. chrysanthemi*. However, the use of enzyme in patients can result in side effects, including thrombotic complications and allergic reactions, which can sometimes make it impossible to continue treatment.^20,21^ Thus, there is a need to explore alternative sources for L-asparaginase production or to develop new engineered forms of the enzyme.

 Protein enzymes are the primary catalysts for almost all biological reactions in all types of life. Moreover, human societies have intelligently utilized these macromolecules for various purposes, such as baking bread, preparing dairy and fermented products, as well as medical applications. In recent times, scientists have gone further to design and engineer new enzymes.^33^ Engineered enzymes are designed for different uses, such as removing undesirable features of natural enzymes or improving features like enzyme activity, substrate specificity, and thermodynamic stability.^34^

 The characteristics of enzymes can be generally classified as catalytic properties, specificity, reversibility of enzyme reactions, and temperature and pH sensitivity. Each characteristic is expressed by a defined, measurable parameter. For example, catalytic activity is represented by kcat, which indicates the number, frequency, or speed of the catalyzed reaction. The Km constant or Michaelis constant is a crucial enzyme constant that is determined through kinetic studies, and it provides insights into several important characteristics of an enzyme, such as the saturation level and substrate affinity.

 Recombinant proteins are produced through biological processes in host cells, and they are called recombinant because the DNA encoding these proteins originates from other species and is recombined or created in the host cells. Insulin was the first recombinant therapeutic protein produced in 1982, and since then, pharmaceutical biotechnology has made tremendous advancements in providing diverse array of products and their quality.^35^

 Our previous study based on bioinformatics and molecular modeling approaches predicted that L-asparaginase enzyme with Y176F/S241C double mutations would improve the thermodynamic stability of the enzyme.^5^ The aim of this study was to express, purify, evaluate the activity, and characterize the new mutant L-asparaginase enzyme. The designed enzyme was produced and purified through recombinant technology and was found to have improved L-asparaginase activity with reduced glutaminase activity compared to the commercial wild type enzyme. The new enzyme can be considered a biosimilar and/or bio-better biological product.

 As mentioned in the results section, three genetic constructs named plasmids A, B and C, were used to produce the mutant enzyme. In construct A, the new mutant L-asparaginase coding gene is in pET-22b ( + ) vector. This construct, which was already available in the lab, was used for protein expression and also was used for subcloning the sequence for new L-asparaginase coding gene in pGEX-6P-1 vector with different designs (plasmids B and C) (Figure 1). In plasmid A, the mutant L-asparaginase coding sequence is preceded by pelB signal sequence, which is expected to direct the expressed enzyme to the periplasmic space. The expression of the new enzyme in various strains of *E. coli* showed that the majority of the produced enzyme was accumulated in the insoluble fraction and likely as the inclusion bodies. Although the presence of the enzyme in the soluble fraction was confirmed through the observation of L-asparaginase activity for the soluble fraction resulting from cell lysis, an attempt to purify the enzyme from the soluble fraction using the 6 × His sequence designed in the gene construct and IMAC chromatography was not successful. The lack of purification of the expressed protein may be due to the fact that the 6 × His sequence is flanked by pelB and the enzyme, thus is not spatially accessible for binding to the nickel-sepharose affinity column. Then, the mutant L-asparaginase gene was subcloned into the expression vector pGEX-6P-1 (Figure 1B) to create a fusion protein with GST, a cleavage site for PreScission protease enzyme, 6 × His sequence, the second cleavage site for factor Xa protease enzyme, and the new mutated L-asparaginase enzyme, respectively. This fusion protein was designed to enable the use of various affinity chromatography methods for the purification step. However, the results indicated the lack of proper expression and purification of the fusion protein. In the next step, plasmid C was prepared to express the GST-PreScission-L-ASPmut fusion protein as shown in Figure 1C. SDS-PAGE analysis of the samples obtained from the expression and purification steps by affinity chromatography with glutathione-sepharose column is shown in Figure 2. The band with a molecular weight of approximately 65 kDa corresponds to the fusion protein GST-PreScission-L-ASPmut. The mutant L-asparaginase enzyme was cleaved off the GST-PreScission-L-ASPmut fusion protein by PreScission protease enzyme, and then was eluted from the column. The band with a molecular weight about 37 kDa corresponds to the new mutated L-asparaginase enzyme (L-ASPmut). The protein obtained from this step was subjected to size-exclusion chromatography for further purification (Figure 3A). The peak related to the enzyme, which has a retention time of 9 minutes, was collected and used for subsequent experiments after concentration determination. For comparison in the next steps, the commercial enzyme was also subjected to size exclusion chromatography under the same chromatography condition (Figure 3B).

 After purification through affinity and size-exclusion chromatography processes, the activity of the designed recombinant mutant enzyme was measured by the Nessler method and compared with the results of the commercial enzyme. According to these results, the specific activity of the new L-asparaginase enzyme (237.8 ± 22.1), which has a pair of Y176F/S241C mutations, is superior to that of the natural *E. coli* L-asparaginase (167.8 ± 22.0). Furthermore, the glutaminase activity of the mutant and commercial enzymes are 17.8 ± 2.5 and 106.6 ± 3.8 IU/mg, respectively, which is in agreement with our previous results,^5^ as well as other reports regarding the glutaminase activity lowering effect of Y176F mutation.^15,26^ Moreover, the kinetic parameters of the mutated L-asparaginase enzyme were evaluated. The production of ammonia over time due to the activity of the mutant enzyme on substrate, shown in Figure 4A, demonstrates that in the early stages of the reaction the enzyme quickly converts asparagine into aspartic acid, releasing ammonia. As the reaction continues, the production of ammonia reaches its maximum, and there are no significant changes in the concentration of the produced ammonia. To determine the kinetic parameters, the initial velocity of the enzyme reaction was measured at different substrate concentrations (asparagine), and the graph of initial velocity (Vo) against substrate concentration [S] was plotted (Figure 4B). The obtained kinetic parameters and other enzyme characteristics for the designed recombinant L-asparaginase enzyme (with Y176F/S241C mutations) are presented in Table 2. The values of Km, Vmax, kcat, and catalytic efficiency of the mutant enzyme are 13.96 mM, 2.218 mM min^-1^, 273.9 min^-1^, and 19.62 mM^-1^ min^-1^, respectively. The values of enzyme parameters for L-asparaginase enzyme from different microorganisms vary widely. For example, Km values, indicating enzyme affinity to the substrate, for L-asparaginase enzymes obtained from different microorganisms have been reported to range from a few micromolar to a thousand millimolar.^31,36-38^ Furthermore, even for the enzyme from a given source, different values are reported in the literature, which can be due to differences in assay conditions such as temperature and pH or substrate and evaluation method. Considering the obtained Km value of approximately 14 mM, it can be concluded that the mutated L-asparaginase enzyme, has a suitable affinity for L-asparaginase. The parameter Vmax represents the maximum rate of the reaction catalyzed by the enzyme when it is saturated by the substrate. The value of Vmax for the mutated enzyme is 2.2 mM/min, which is comparable to the range of several micromolar to several millimolar/min reported for the L-asparaginase enzyme in different sources.^31^ To compare the properties of the mutated enzyme with the reported values, it is necessary to consider the concentration of the enzyme. As the Vmax value depends on the concentration of the enzyme, it is possible to compare or normalize the properties in terms of concentration units of the enzyme. The kcat, specific activity and catalytic efficiency determined for the mutant enzyme are concentration curated values and can be compared to those reported for other L-asparaginases reported in the literature.

**Table 2:** Kinetic properties of recombinant L-asparaginase

**Purification step**	**Total protein (mg)**	**Total activity (U)**	**Specific activity (U/mg)**	**Recovery (%)**	**Purification yield (Fold)**
Soluble fraction	973.70	2973.3	3.1	100	1
Sample from AC	1.70	99.5	58.6	3.4	18.9
Sample from SEC	0.09	21.4	237.8	0.7	76.7
**Enzyme kinetic parameters**					
	**Km (mM)**	**Vmax (mM/min)**	**kcat (min**^-1^**)**	**Specific activity (IU/mg)**	**Catalytic efficiency (mM**^-1^** min**^-1^**)**
	13.96	2.218	273.9	237.8	19.62

 Three-dimensional structure of a protein is stabilized by the interplay of various stabilizing and destabilizing forces. For example, limiting the available conformations for the protein chain in the folded state through the reduction of entropy has a negative effect on folding, while ionic, van der Waals and hydrogen bond interactions reduce ∆G through negative enthalpy effects and have positive effect on protein stability. The thermodynamic stability or changes in the folding free energy (∆G) of a protein are expressed in relation to the concentration ratio of the folded [F] and denatured [U] states, according to the equation ∆G = -RT Ln ([F]/[U]). The conversion of protein chain into a folded state under proper condition in terms of temperature, pH, salt and etc. is a spontaneous reaction with a favorable negative free energy value. Any external factor that directs the [U] ↔ [F] balance towards denaturation causes ∆G to become more positive and consequently leads to structural instability of the protein. In the process of protein folding, hydrophobic interactions play a major role. The process of protein folding involves the arrangement of hydrophobic side chains in a manner that they move away from water and interact with other hydrophobic side chains within the protein’s interior. One method of protein denaturation includes adding chaotropic chemical compounds, such as urea, guanidinium chloride, thiourea, and magnesium chloride, to the protein solution. These compounds increase ∆G, leading to protein denaturation due to the reduction of hydrophobic interactions.

 To investigate the stability energy of the new L-asparaginase enzyme, its intrinsic fluorescence was examined in the presence of varying concentrations of guanidinium chloride (GdmCl). The intensity and characteristics of intrinsic fluorescence in folded and denatured proteins are different, allowing for the determination of the ratio of F and U forms under different GdmCl concentrations. Figure 5A illustrates the changes in the intrinsic fluorescence spectrum of mutated L-asparaginase enzyme in the presence of 0 to 6 M GdmCl. Low concentrations of GdmCl (0 to 1 M) seem to cause dissociation of homo tetrameric quaternary structure of the enzyme (Figure 5B). At higher concentrations, GdmCl denatures the subunits, and the pattern of changes in the fluorescence spectrum (Figure 5C) is distinct from that at low GdmCl concentration phase. To assess the stability energy of the L-asparaginase enzyme monomers, changes in fluorescence intensity at the maximum emission wavelength of 307 nm were analyzed. Figure 6 illustrates the fraction of the folded form of L-asparaginase enzymes against GdmCl concentrations. The data shows that compared to the wild type, a slightly higher GdmCl concentration is required to unfold the mutant L-asparaginase. The ratio of [U] to [F] is used to calculate ∆G of unfolding, as presented in Figure 7. Based on these results, it can be deduced that the stability of the L-asparaginase enzyme with Y176F/S241C mutations is higher (8421 J/mol) than that of the commercial L-asparaginase enzyme (5274 J/mol), which is consistent with our previous *in silico* study.^5^

## Conclusion

 The L-asparaginase enzyme catalyzes the conversion of amino acid L-asparagine into L-aspartic acid and ammonia, and is considered an essential pharmaceutical agent in the treatment of ALL in children. Currently, the clinically used L-asparaginase is produced via biotechnology methods based on the enzyme sequences of *Escherichia coli* and *Erwinia chrysanthemi* microorganisms. However, the use of this medication causes some side effects, such as allergic reactions, which sometimes make it impossible to continue the treatment. Consequently, the development of L-asparaginase from new sources or introducing novel engineered enzymes to produce alternative products have long been considered. In this study, some of the characteristics of an engineered *E. coli* L-asparaginase containing Y176F/S241C mutations were determined. The pure mutant L-asparaginase was obtained by affinity and size-exclusion chromatography methods. Despite a purification efficiency of approximately 77 folds, the recovery of the mutant enzyme was very low and below 1%. The mutant enzyme showed higher asparaginase and lower glutaminase activities compared with wild type enzyme. Different enzymatic parameters were determined for the catalysis of asparagine to aspartic acid conversion by the mutant enzyme. The values of Km, Vmax, kcat, and catalytic efficiency of the new enzyme were determined to be 13.96 mM, 2.218 mM min^-1^, 273.9 min^-1^, and 19.62 mM^-1^ min^-1^, respectively. The folding stability of the mutant L-asparaginase enzyme was investigated via chemical denaturation method using GdmCl as the denaturing agent. The free energy of unfolding for the mutant and wild type enzyme were 8421 and 5274 J/mol, respectively, indicating the higher stability of the mutant enzyme compared with the wild type enzyme. This finding is consistent with the previously reported *in silico* results.

## Acknowledgments

 Authors would like to thank Research Office and Biotechnology Research Center of Tabriz University of Medical Sciences for providing financial support and laboratory facilities.

## Competing Interests

 The authors declare no conflict of interest

## Ethical Approval

 This article does not involve any study with human participants or animals to be performed by any of the authors.

## Supplementary Files

Supplementary file 1 contains Figure S1 and S2.
