# Supplementary material for: Investigating Functional and Folding Stability of an Engineered E. coli L-asparaginase Harboring Y176F/S241C Mutations
Source: Adv Pharm Bull. 2024 Jun 22;14(3):675–85. doi: 10.34172/apb.2024.048 (PMC11530880; doi:10.34172/apb.2024.048)
Supplement: Supplementary file 1 — contains Figure S1 and S2. [file apb-14-675-s001.pdf]

Supporting information

Doi: 10.34172/apb.2024.048

Investigating Functional and Folding Stability of an Engineered *E. coli* L-asparaginase Harboring Y176F/S241C Mutations

Mahrokh Dastmalchi[1,2], Maryam Hamzeh-Mivehroud[2,3], Hassan Rezazadeh[3,4], Mohammad M Farajollahi[1], Siavoush Dastmalchi[2,3,5]*

[1]Department of Medical Biotechnology, Faculty of Allied Medical Sciences, Iran University of Medical Sciences, Tehran, Iran
[2]Biotechnology Research Center, Tabriz University of Medical Sciences, Tabriz, Iran.
[3]School of Pharmacy, Tabriz University of Medical Sciences, Tabriz, Iran.
[4]Pharmaceutical Analysis Research Center, Tabriz University of Medical Sciences, Tabriz, Iran.
[5]Faculty of Pharmacy, Near East University, POBOX:99138, Nicosia, North Cyprus, Mersin 10, Turkey.

Correspondence: Siavoush Dastmalchi, Emails: dastmalchi.s@tbzmed.ac.ir; siavoush11@yahoo.com

supplementary

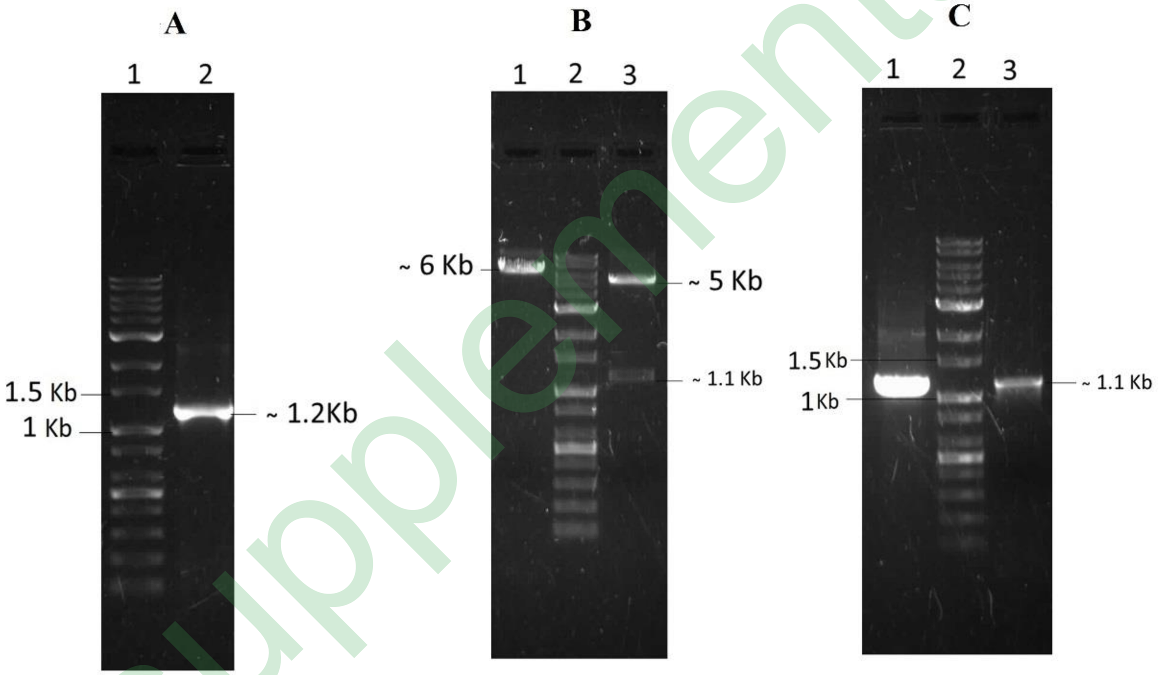

Figure S1. Results of PCR reactions illustrated by agarose gel electrophoreses. A) The result of electrophoresis on the PCR product using plasmid C as the DNA template and universal primers for pGEX vectors. The band about 1.2 Kb corresponds to the amplified gene coding for mutant L-asparaginase and flanking segments. B) The results of electrophoresis on single (*Bam*H1) and double digested (*Bam*H1 and *Eco*R1) plasmid B are shown in lanes 1 and 3, respectively. The digestion patterns are in agreement with the intended construct, i.e., plasmid B. C) Lane 1 is the sample prepared from PCR reaction using plasmid A as the DNA template and F1 and R pair of primers. Lane 2 shows the result of electrophoresis on the sample prepared from PCR reaction where the same set of primers (F1 and R) and bacterial colony transformed by plasmid B were used in the reaction. The band about 1.1 Kb in lanes 1 and 3 confirms the correctness of the plasmid B.

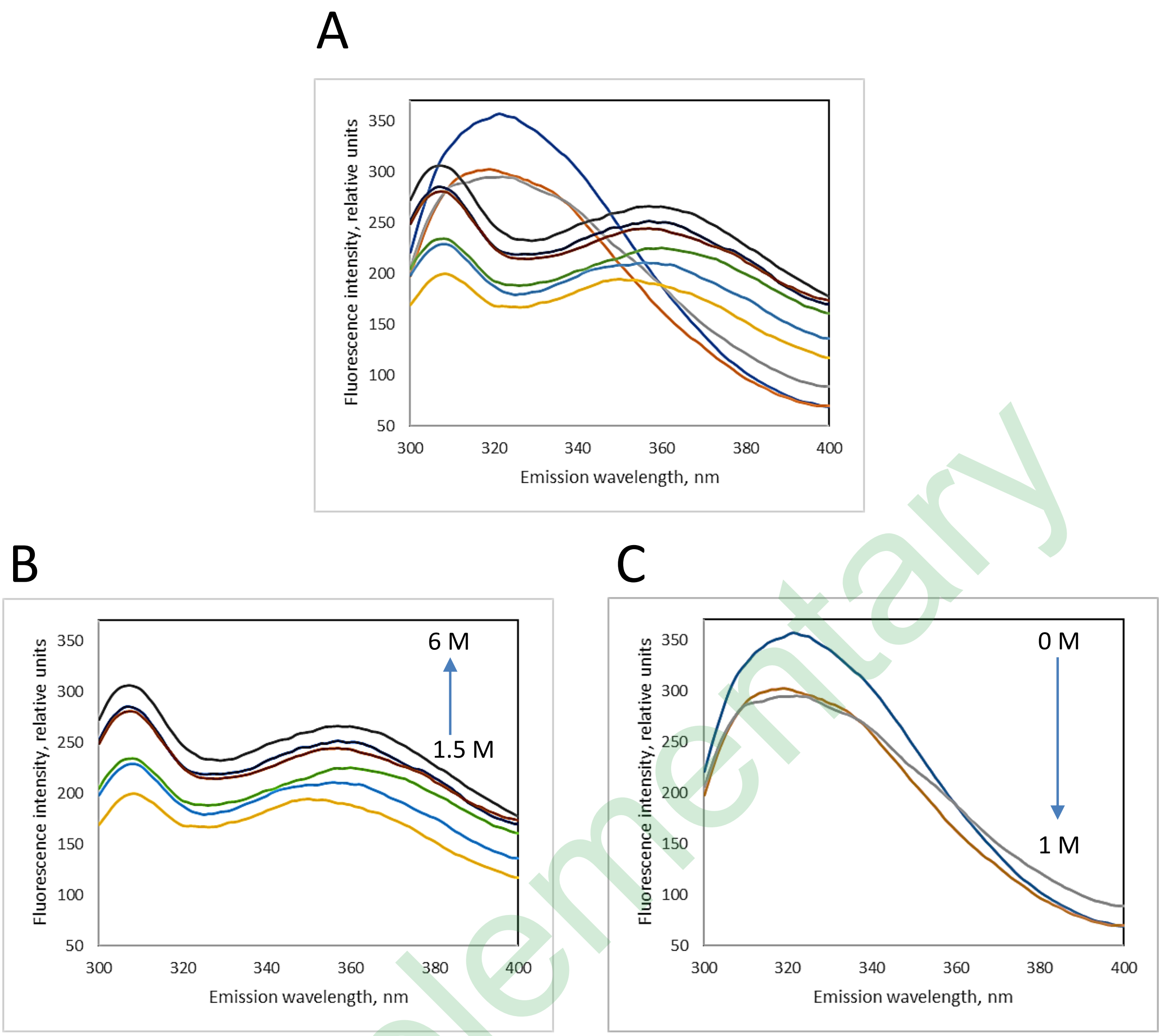

Figure S2. (A) Fluorescence emission spectra of SEC purified commercial L-asparaginase in the presence of different concentrations of guanidinium chloride (0 to 6 M). Due to changes in the pattern of fluorescence spectra at different concentration of GdmCl, they are separated in two graphs B and C.

supplementary
